# The IUPHAR/BPS guide to PHARMACOLOGY in 2022: curating pharmacology for COVID-19, malaria and antibacterials

**DOI:** 10.1093/nar/gkab1010

**Published:** 2021-10-30

**Authors:** Simon D Harding, Jane F Armstrong, Elena Faccenda, Christopher Southan, Stephen P H Alexander, Anthony P Davenport, Adam J Pawson, Michael Spedding, Jamie A Davies, NC-IUPHAR

**Affiliations:** Deanery of Biomedical Sciences, University of Edinburgh, Edinburgh EH8 9XD, UK; Deanery of Biomedical Sciences, University of Edinburgh, Edinburgh EH8 9XD, UK; Deanery of Biomedical Sciences, University of Edinburgh, Edinburgh EH8 9XD, UK; Deanery of Biomedical Sciences, University of Edinburgh, Edinburgh EH8 9XD, UK; School of Life Sciences, University of Nottingham Medical School, Nottingham NG7 2UH, UK; Experimental Medicine and Immunotherapeutics, University of Cambridge, Cambridge CB2 0QQ, UK; Deanery of Biomedical Sciences, University of Edinburgh, Edinburgh EH8 9XD, UK; Spedding Research Solutions SAS, Le Vésinet 78110, France; Deanery of Biomedical Sciences, University of Edinburgh, Edinburgh EH8 9XD, UK

## Abstract

The IUPHAR/BPS Guide to PHARMACOLOGY (GtoPdb; www.guidetopharmacology.org) is an open-access, expert-curated database of molecular interactions between ligands and their targets. We describe expansion in content over nine database releases made during the last two years, which has focussed on three main areas of infection. The COVID-19 pandemic continues to have a major impact on health worldwide. GtoPdb has sought to support the wider research community to understand the pharmacology of emerging drug targets for SARS-CoV-2 as well as potential targets in the host to block viral entry and reduce the adverse effects of infection in patients with COVID-19. We describe how the database rapidly evolved to include a new family of Coronavirus proteins. Malaria remains a global threat to half the population of the world. Our database content continues to be enhanced through our collaboration with Medicines for Malaria Venture (MMV) on the IUPHAR/MMV Guide to MALARIA PHARMACOLOGY (www.guidetomalariapharmacology.org). Antibiotic resistance is also a growing threat to global health. In response, we have extended our coverage of antibacterials in partnership with AntibioticDB.

## INTRODUCTION

The Guide to PHARMACOLOGY (shortened to GtoPdb) is jointly developed by The International Union of Basic and Clinical Pharmacology (IUPHAR) and the British Pharmacological Society (BPS) and originated from the former IUPHAR-DB, a resource focused on receptors and channels that was first compiled in 2003 (1–3), and from the BPS ‘Guide to Receptors and Channels’ (4), a compendium, providing concise overviews of the key properties of a wider range of targets than those covered in IUPHAR-DB. These two resources were first merged in 2011. The combined resource continued to expand its coverage of target families and quantitative target-ligand interactions with expert guidance and oversight maintained through the Nomenclature and Standards Committee of the International Union of Basic and Clinical Pharmacology (NC-IUPHAR) together with its 109 subcommittees, comprising over 1000 scientists (5). The database scope expanded to cover the data-supported druggable human genome (6); immunopharmacology through the Wellcome-trust funded IUPHAR Guide to IMMUNOPHARMACOLOGY (GtoImmuPdb; https://www.guidetoimmunopharmacology.org) (7–9); and, most recently, malaria pharmacology through the Guide to MALARIA PHARMACOLOGY (GtoMPdb; www.guidetomalariapharmacology.org) (10), a collaboration between IUPHAR and Medicines for Malaria Venture (MMV; www.mmv.org).

Since our last update in 2020 (10), our curatorial effort has focused on three main areas. First, we have continued to work closely with MMV to extend the Guide to MALARIA PHARMACOLOGY. Second, a more recent collaboration with AntibioticDB (ADB; www.antibioticdb.com) has brought added value to both resources, providing chemistry and pharmacology information for antibacterials curated in ABD and in turn expanding GtoPdb content to clinically useful antibacterials. Finally, but perhaps most significantly, as a rapid response to the SARS-CoV-2 pandemic we applied our expertise to capture and evaluate the rise in pharmacological strategies being considered to combat SARS-CoV-2 infection and/or COVID-19 symptoms. In this paper we describe recent extensions to the database and our most recent website modifications, including new ways to access and download data and new links to external resources that bring added value.

## GUIDE TO PHARMACOLOGY UPDATES

### Summary of database content and curation

Curation and database development is conducted by the GtoPdb Curation Team (www.guidetopharmacology.org/about.jsp#curation) based at The University of Edinburgh and is overseen by NC-IUPHAR (2). Our unique approach to curation involves expertise at all stages. Data are selected by NC-IUPHAR subcommittees, comprising ∼1000 scientists worldwide, and cover established drug targets and those of emerging interest for drug discovery. GtoPdb's strength lies in this considered, independent and expert-led curation and the prioritisation of data validated from independent sources. A summary of targets, ligands and interactions curated in the latest release of GtoPdb (v2021.3, 2 September 2021) is shown in Table 1. For all ligands, targets and target families referred to in this paper, GtoPdb IDs and URLs to GtoPdb entry pages can be found in supplementary data Supplementary table S1.

**Table 1.** Guide to PHARMACOLOGY data counts for targets, ligands and interactions from database release 2021.3. The changes from the 2019.4 (September 2019) release are shown in parenthesis

**A. Target class content. Human UniProtKB accession counts**	
GPCRs	399 (+1)
Nuclear hormone receptors	48 (0)
Catalytic receptors	253 (+4)
Ion channels	278 (0)
Transporters	555 (+16)
Enzymes	1246 (+34)
Other proteins	216 (+9)
Total number of targets	2995 (+63)
**B. Ligand category counts**	
Synthetic organics	7593 (+1069)
Metabolites	516 (–69)
Endogenous peptides	803 (+13)
Other peptides including synthetic peptides	1421 (+83)
Natural products	334 (+68)
Antibodies	317 (+56)
Inorganics	39 (0)
Approved drugs	1688 (+246)
Withdrawn drugs	88 (+17)
Coronavirus	82
Antibacterials	303
WHO essential list	282 (+89)
Antimalarial	114 (+42)
Ligands with INNs	2884 (+531)
PubChem CIDs	8262
PubChem SIDs	11 025
Total number of ligands	11 025 (+1222)
**C. Interaction counts**	
Human targets with ligand interactions	1847 (+66)
Human targets with quantitative ligand interactions	1596 (+71)
Human targets with approved drug interactions	674 (+36)
Primary targets* with approved drug interactions	335 (+6)
Ligands with target interactions	9224
Ligands with quantitative interactions (approved drugs)	8161 (+706) 1018 (+93)
Ligands with clinical use summaries (approved drugs)	3005 (+569) 1684 (+245)
Number of binding constants	49 831 (+1300)
References	41 041 (+4601)

^a^Primary target indicates the dominant Molecular Mechanism of Action (MMOA). The table includes a comparison to the figure in the 2020 update (10). Categories are not mutually exclusive, and targets and ligands can fall into more than one.

### Ligands

Since our last update, we have added 1222 new ligands as summarised in Table 2. The categories shown reflect areas of curatorial expansion for coronavirus, antibacterials and antimalarials, and compares total ligand counts from our previous update (10). It also indicates where existing ligands, already curated in GtoPdb, have been updated to be included in these categories. For example, for ligands relevant to COVID-19, 28 new compounds were added, but a further 54 existing ligands in GtoPdb were marked as COVID-19 relevant. In addition to COVID-19 relevant ligands we have also added 190 approved drugs, 55 WHO essential medicines, 34 antimalarial compounds and 280 antibacterial compounds. Overall, around 50% (674) of new ligands have quantitative interaction data.

**Table 2.** Summary of new ligands added to GtoPdb in the 2021.3 database release with comparison to the 2019.4 release (September 2019). ‘New Ligands’ column shows count of new ligands for each category; ‘Updated Ligands’ shows count of existing ligands, already curated in GtoPdb, now included in the categories. Column 4 and 5 shows the total ligands count for each category from our 2021.3 (September 2021) and 2019.4 (September 2019) database releases

	New ligands	Updated ligands	Total ligands (2021.3)	Total ligands (2019.4)
Approved drugs	190	56	1688	1442
WHO essential medicines	55	34	282	193
Antibacterials	280	23	303	0
Ligands with quantitative interaction data	679	27	8161	7455
Antimalarials	37	5	114	72
COVID-relevant ligands	28	54	82	0
All ligands	1222	0	11 025	9803

### Targets

Targets in GtoPdb use a UniProtKB/SwissProt (11) accession as their primary identifier and are organized into hierarchical target families. Table 1A shows the count of targets curated in GtoPdb against different top-level target classes, showing a total of 2995 targets, an increase of 63 since our last update. In fact, there are 90 new targets added to GtoPdb, but 27 of these are either *Plasmodium* targets or coronavirus proteins and therefore lack the Human UniProtKB identifiers used in our counts.

Interaction data are at the heart of GtoPdb, and Table 1C shows that there is at least one curated ligand interaction for 1847 human protein targets, 1596 of which have quantitative binding data. Restricting this analysis of human targets with quantitative binding data to approved drugs shows 674 interactions, 335 of which are where the protein is the primary target of the drug.

### Coronavirus pharmacology

As the emergence of SARS-CoV-2 developed into a pandemic, and the symptoms of COVID-19 became clearer, it was important for IUPHAR, through the GtoPdb, to apply its expertise and rigorous scientific principles to evaluate the surge in pharmacological strategies (including drug repurposing efforts) and molecular mechanisms that were being considered to combat SARS-CoV-2 infection and/or COVID-19 symptoms (12).

In response, a new coronavirus information page was set-up on the GtoPdb website (www.guidetopharmacology.org/coronavirus.jsp), with the content providing the impetus for the publication of ‘A rational roadmap for SARS-CoV-2/COVID-19 pharmacotherapeutic research and development: IUPHAR Review 29’ (13). This page is updated weekly (compared to quarterly for the main website) to allow rapid dissemination of reviewed and curated coronavirus therapeutic developments. Tables of agents (now >100) that have verified activity, and both established and emerging host and coronavirus targets, are regularly reviewed and updated with detailed curator comments and links to pharmacological data within the GtoPdb. They include out-links to primary literature and other external resources, such as clinical trials (at https://clinicaltrials.gov), drug company press releases and pre-prints (we routinely update our database when peer-reviewed versions become available).

Medicinal chemistry and biochemistry journals are the primary sources of new ligands. This is supplemented by notifications (from trusted sources) on social media platforms, pre-prints, and drug company press releases. The lists of proposed International Non-proprietary Names (INNs) from the WHO have also proven to be a rich source of information. Two lists of proposed INNs specific for COVID therapeutics have been published since October 2020, and these have included INNs for vaccines, anti-spike monoclonal antibodies, small molecule antivirals, and agents with other mechanisms of action. It was possible to identify sufficient information for 16 of the 25 proposed INNs in list 124 (COVID special) to meet GtoPdb curation criteria. Small molecules are mapped to chemistry databases using their SMILES (Simplified Molecular Input Line Entry System), which can be computationally generated from the IUPAC descriptors in the INN document using the free online tool OPSIN: Open Parser for Systematic IUPAC nomenclature (https://opsin.ch.cam.ac.uk/). In a number of cases, the chemical structures are traced to patents which often contain interaction data that has not been published in peer-reviewed form. Examples of GtoPdb entries for antivirals that have been created using this method include bemnifosbuvir, which was identified as Atea Pharmaceuticals’ AT-527, an HCV RNA polymerase inhibitor that has been redeployed for anti-SARS-CoV-2 activity, lufotrelvir (GtoPdb ID: 11249) which mapped to Pfizer's Mpro (SARS-CoV-2 Main Protease) inhibitor PF-07304814, and molnupiravir which mapped to the clinical stage RNA-dependent RNA polymerase inhibitor EIDD-2801/MK-4482. The receptor-interacting protein kinase inhibitor eclitasertib and the Toll-like receptor 7/8 antagonist enpatoran, both of which are proposed to have anti-inflammatory activities, were also curated using this strategy.

The GtoPdb now includes interaction data for > 40 Mpro inhibitors, including those that are in clinical trials (e.g. Pfizer's oral clinical lead PF-07321332). Eleven anti-spike monoclonal antibodies, including imdevimab and casirivimab, which are the active ingredients in Regeneron's cocktail Ronapreve, which was approved for use in the UK in August 2021) are now curated in the GtoPdb.

Searching GtoPdb with the terms SARS-CoV-2 or COVID-19 generates more extensive lists of ligands and targets (201 results and 128 results, respectively) that have coronavirus-related curation, but which may not meet the standards required to merit inclusion on the ‘Coronavirus information’ page. Those drugs or other therapeutics (e.g. monoclonal antibodies) approved for clinical use in COVID-19 patients by national agencies are posted at the top of the ‘Ligands’ tab on the information page. The page also provides a resource where the GtoPdb can host data and other evidence that shows that certain drugs have failed to produce clinical efficacy.

### Coronavirus proteins

A new family of Coronavirus (CoV) proteins was created to allow the curation of ligand → target interactions and associated pharmacological data for these non-mammalian proteins. The family is included as part of the ‘anti-infective targets’ family (within ‘Other proteins’), which was originally added to accommodate the *Plasmodium* targets.

Small molecules being developed as potential SARS-CoV-2 therapeutics have targeted the limited number of virus proteins with precedents for activity modulation. These include the 3CL-like/main protease (3CLpro or Mpro), papain-like protease (PLpro) and RNA-dependent RNA polymerase (RdRp; the molecular target of remdesivir. The distinct individual Mpro, PLpro and RdRp enzyme sequences are mapped to published inhibitors screened against these isolated enzymes *in vitro* and their MERS-CoV and SARS-CoV orthologues (all of which now have representative RCSB Protein Data Bank structures (www.rcsb.org/). Note that for each of these targets, the ligands can be downloaded as .csv files.

Most other databases have opted to map their curated inhibitors to the 7096-aa polyprotein entry UniProtKB QHD43415 (https://cdsouthan.blogspot.com/2016/02/zika-and-other-flavivirus-polyproteins.html). However, this can result in a confusing concatenation of inhibitor mappings to the polyproteins rather than the distinct enzymes released from these precursors in vivo.

The GtoPdb Coronavirus page is included as one of the BPS’s COVID-19 trusted resources (www.bps.ac.uk/covid-19/resources-and-trusted-information/journals-and-publications), as well as on the European Data COVID-19 Data Portal (www.covid19dataportal.org/related-resources) and at both the ELIXIR (https://elixir-europe.org/services/covid-19#access) and ELIXIR-UK (https://elixiruknode.org/elixir-uk-our-support-to-covid-19-research/) data hubs.

### Antibiotic DB collaboration

GtoPdb now collaborates with Professor Laura Piddock and her research group at Antibiotic DB (ADB; www.antibioticdb.com). Through this interaction, GtoPdb provides chemistry and pharmacology for the antibacterial compounds curated within ADB. Compound names used in ADB were mapped to PubChem CIDs via the Identifier Exchange Service (https://pubchem.ncbi.nlm.nih.gov/idexchange/idexchange.cgi) and the SMILES from PubChem were used to generate the chemical structures for the ligand entries in GtoPdb where there were mappings. Where no name > PubChem mapping could be determined, the curation depended on literature searches, mapping back to PubChem using the SMILES where chemical structures could be identified. Since GtoPdb has an ongoing *modus operandi* of including approved drugs, the curation effort included assessing both USA (Drugs.com, an efficient route into US approved antibacterials; www.drugs.com/drug-class/anti-infectives.html) and UK (British National Formulary; https://bnf.nice.org.uk/) approval resources, and the current WHO Essential Medicines list (www.who.int/publications/i/item/WHOMVPEMPIAU2019.06), as part of the process to include as many antibacterials approved for clinical use as possible. The focus of ADB has traditionally been the development of novel antibacterials, so their approved drug content was not as comprehensive compared to the number of antibacterials approved for clinical use that were added to GtoPdb. This synergy has benefitted users of both databases.

Each new antibacterial ligand entry in GtoPdb was manually curated to include external database links, mechanism of action information, clinical approval status, and bioactivity data relating to the antibacterial potency of the ligand against pathogens. Identifying the primary literature descriptions for many of the older antibacterial compounds was time consuming, and often where the original article could be found, these did not include conclusive chemical structure information.

The ADB team has spent considerable time updating their database in response to issues arising during the GtoPdb curatorial review. For example, many of their original entries were for mixtures, or sets of related chemical analogs, whereas the GtoPdb depends upon discrete chemical structures of each of its ligand entries. The ADB team generated new entries for each component of mixtures where possible.

As indicated in Table 1 there are 303 ligands now tagged in GtoPdb as ‘antibacterial’ as a result of this curatorial effort, most of which are new ligands added since our last update (Table 2). Beyond the curatorial review described above, the collaboration has also brought added value by setting up direct links between GtoPdb ligand summary pages and ADB compound records, which currently stands at 246 links from 230 ligands. The discrepancy between linked and tagged ligands is explained by ADB’s focus on novel or development compounds for unmet clinical need, whereas GtoPdb has tried to include all approved antibiotics.

### Malaria pharmacology

As described previously (10), the IUPHAR/MMV Guide to MALARIA PHARMACOLOGY (GtoMPdb) has been developed as an extension to the main GtoPdb database, with the aim of providing optimized access for the malaria research community to the data in GtoPdb. The resource was officially launched in September 2019 and is available at www.guidetomalariapharmacology.org

Since the first public release, expansion of GtoMPdb content has continued. The latest database release (v2021.3, 2 September 2021), includes 114 curated ligands tagged as ‘antimalarial’ and 39 *P. falciparum* (3D7) targets (a more detailed summary of targets, ligands and interactions is provided in Table 3).

**Table 3.** Guide to MALARIA PHARMACOLOGY data counts for targets, ligands and interactions from database release 2021.3

**Guide to Malaria Pharmacology Content Breakdown**	
Targets	39
Ligands	114
Ligands (approved drugs)	19
Ligands (WHO essential lists)	17
Targets with quantitative ligand interactions	31
Targets with approved drug interactions	5
Ligands with interactions	107
Ligands with interaction to known targets	68
Ligands with interaction to unknown targets	46
Ligands with quantitative interactions (approved drugs)	104 (18)
Ligands with clinical use summaries (approved drugs)	48 (24)

The ability to search across GtoMPdb for malaria data has also been extended. We have added the ability to search via PlasmoDB identifiers on our general search tools and have extended our web services to enable filtering on malaria targets and ligands. The web services (www.guidetopharmacology.org/webServices.jsp#interactions) can also be used to bring back specific interactions for each *Plasmodium* species and this will extend in the future to filtering on the malaria parasite's lifecycle stages.

### Extension of target hierarchy for malaria

Recent work has focussed on the addition of target subfamilies to the Antimalarial targets family. This new classification is still under review but has helped categorize and organize the targets into meaningful groups and allowed additional information to be added as overviews on the subfamily pages. These pages will continue to be developed following feedback from members of the Malaria Drug Accelerator (MalDA), a consortium working to expedite the development of new antimalarial medicines by identifying new druggable targets and early lead inhibitors (14). Our work with MalDA will form the basis of an IUPHAR Review on recent advances in malaria pharmacology and the GtoMPdb resource (manuscript in preparation).

### Using GtoPdb

Detailed help and tutorials are provided on our website and we have previously written a protocol paper about accessing data in GtoPdb (15), and for the IUPHAR Guide to Immunopharmacology, we have described a use-case on how the resource can support research in vascular inflammation (9).

The open-access nature of GtoPdb, along with is expert-backed curation, makes it an ideal resource for easily accessing quantitative binding data to help answer research questions. For example, in 2018 Siafis and Papzisis addressed the important question of why some antidepressants have been associated with development of diabetes (16). They used GtoPdb and the PDSP (Psychoactive Drug Screening Program) Ki database (17) to retrieve quantitative pharmacodynamic data for a set of 22 FDA-approved antidepressants. These data were used to illustrate the occupancy of antidepressants on human transporters and receptors (see Figure 2 in the reference), and to seek evidence for any correlation between binding to particular targets and risk of pro-diabetes activity. The authors' analysis suggested that higher degrees of occupancy on H_1_ and muscarinic receptors, except for M_2_, seem to be related with higher risk for diabetes. This potentially gives an ‘early warning’ for this type of side-effect (if a new drug binds these, be especially careful of this possible side-effect).

### Website & interface updates

#### Site usage

GtoPdb is an open-access and free resource that aims to provide accurate information on the basic science underlying drug action to support research and education. It continues to be very well accessed by users, which we can track via Google Analytics. In the 18 months to 31st July 2021 the site has seen a monthly average of ∼43 400 sessions from ∼28 300 users and an average of 141 000 page views per month. Specifically our coronavirus information page (www.guidetopharmacology.org/coronavirus.jsp) has been accessed around 30 000 times since April 2020.

We can also monitor specific subsets of data, such as those curated as part of the Guide to Malaria Pharmacology. When we analyse the page view counts we can see that, for all antimalarial targets, the detailed view pages have been viewed on average 128 times per month and antimalarial ligand pages viewed on average 830 times per month. Taking all malarial pages into account, the GtoMPdb data are viewed around 1500 times per month.

**Figure 1.**
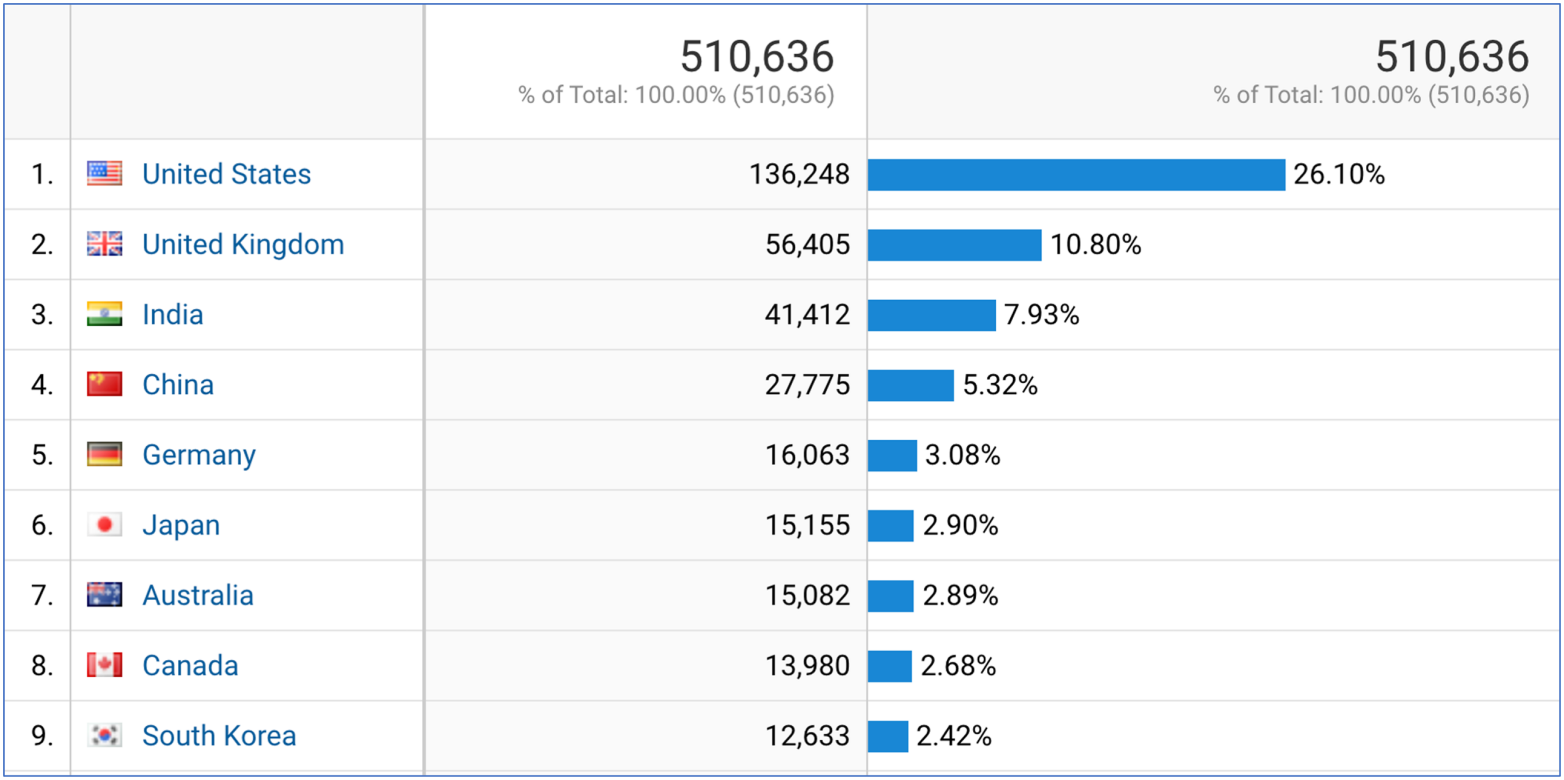
Google Analytics chart showing number of users accessing GtoPdb by country of origin from February 2020 to July 2021. Limited to those countries with over 10,000 total users during the time period.

More broadly, although access to GtoPdb is dominated by the UK and USA (∼36% of users), access comes from across the globe. In the 18 months to 31 July, a total of 225 different countries recorded at least one user, 57 countries recorded 1000 or more users and 9 countries recorded over 10 000 users (US, UK, India, China, Germany, Japan, Australia, Canada, South Korea), see Figure 1.

#### Ligand summary pages

**Figure 2.**
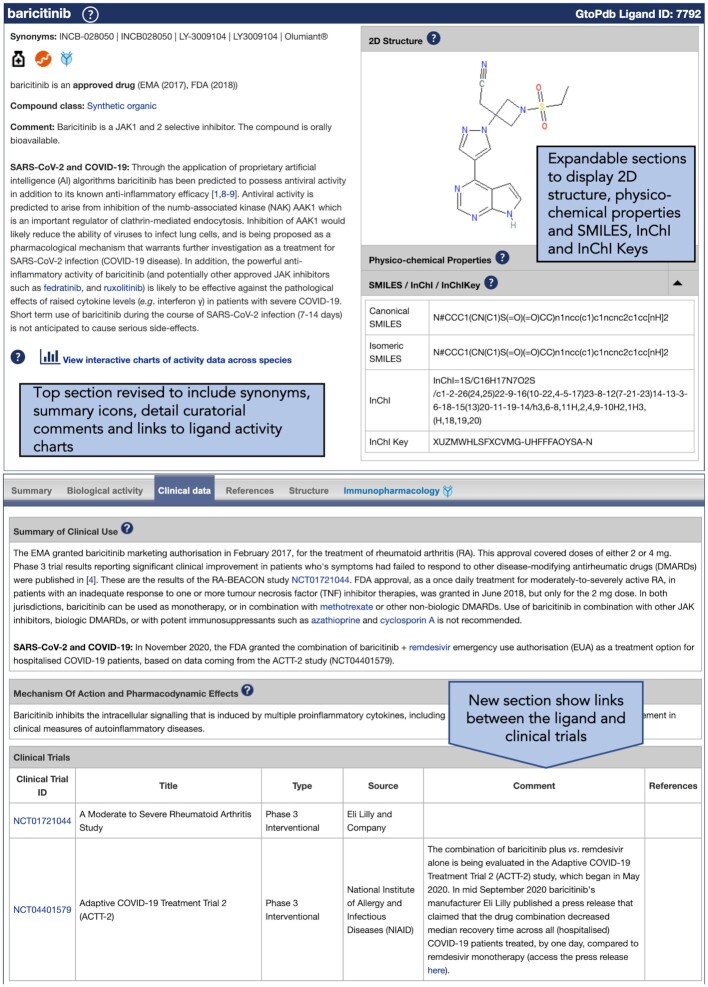
Example of the main updates to GtoPdb Ligand Summary Pages, here showing the ligand baricitinib (www.guidetopharmacology.org/GRAC/LigandDisplayForward?ligandId=7792).

Our ligand summary pages have been revised to place the key information at the top of the page. In Figure 2, we show how this looks for baricitinib. The top section now includes synonyms, icons to indicate key ligand classifications, and drug approval status. The curatorial comments are also now presented here. These contain valuable descriptions of the ligand, manually-curated expert information on the ligand's pharmacology, and explain why they have been curated in GtoPdb.

In addition, the 2D ligand structure is displayed beside this information in an expandable section where users can also view physico-chemical properties and SMILES/InChI/InChI Keys.

#### Clinical trial data

Curation of data from clinical trials has always been part of the GtoPdb curation protocol, but reference to trial data that, until recently, had only been recorded in free-text clinical use comments. We have extended the database to curate these data more formally and directly link to clinical trials, ideally with an identifier, for our ligands. These data are now shown on the ligand summary pages (Figure 2), with curated clinical trials involving the ligand displayed in a separate table under the clinical data tab. The table includes links out to the clinical trial plus trial title, type, source, curator comments and references. We anticipate that this adaptation will aid researchers in translational pharmacology.

#### Ligand activity charts

GtoPdb has previously made available ligand activity charts as a tool to summarise pharmacological parameters across species. Their aim is to make finding these data easier, as they may often be reported in the results section of papers and may not appear in the summary, so cannot be easily found in databases such as PubMed (https://pubmed.ncbi.nlm.nih.gov/). The charts display data across species, where these data exist for ligands in GtoPdb and ChEMBL (18) (www.ebi.ac.uk/chembl/). This is useful for researchers trying to identify tool compounds and to calculate concentrations required to achieve a specific pharmacological action. Additionally, knowing species differences can be particularly useful for translational research, and whether ligands are selective or activities at other targets have been reported.

**Figure 3.**
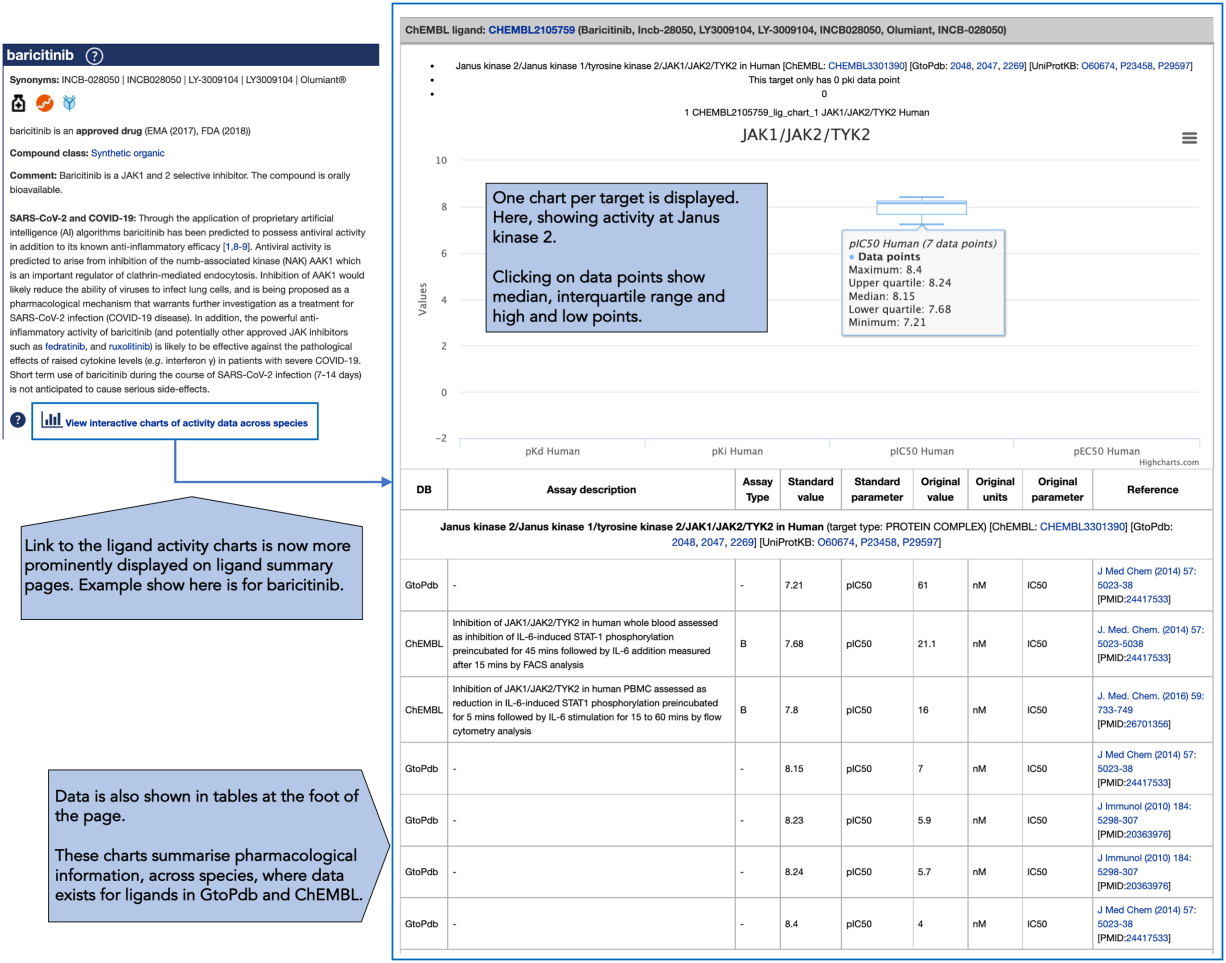
GtoPdb ligand activity charts, showing example for baricitinib. The box plot charts are accessed from ligand summary pages and show activity data from GtoPdb and ChEMBL. Data are also presented in tabular format.

The reorganization of the ligand summary pages has therefore been a good opportunity to make the ligand activity charts more accessible. The top section now contains a direct link to the charts where data are available, an example of which is shown in Figure 3. In addition, a new icon is also displayed on the ligand list pages, which indicates whether there are activity charts available for the ligand and links through to the charts.

The charts themselves are box plots that summarise all of the activity data for a ligand taken from ChEMBL and GtoPdb across multiple targets and species. Separate charts are created for each target, and where possible, the algorithm tries to merge ChEMBL and GtoPdb targets by matching them on name and UniProtKB accession for each available species. However, please note that infrequent inconsistencies in the naming of targets may lead to data for the same target being reported across multiple charts.

#### Downloads

Provision of data in simple reusable formats is essential in helping to ensure GtoPDB is FAIR-compliant (Findable, Accessible, Interoperable, Reusable), for which all public data resources should be striving (https://fairsharing.org/;https://fairsharing.org/FAIRsharing.f1dv0). It offers users a valuable way to access and investigate GtoPdb data.

The GtoPdb download page (www.guideopharmacology.org/download.jsp) provides comma-separated and tab-delimited files (compatible with multiple spreadsheet packages) of GtoPdb targets, ligand, interactions and more. Across the site, we have also added additional buttons to allow users to download specific data sets. On average, data files are downloaded from GtoPdb ∼210 times a month. We also make available our PostgreSQL dump file of the entire database, which has been downloaded 87 times in the past year. A specific file of coronavirus ligands has been downloaded ∼320 times since it was first made available in April 2020.

#### Ligand download

We have expanded the ability to download ligand data from GtoPdb by including download links on our ligand list pages (www.guidetopharmacology.org/GRAC/LigandListForward?database=all). Different categories or groups of ligands can be selected via tabs at the top of the ligand list page. These lists can now be downloaded directly in CSV format. We have also added new ligand download files to our download page. The first is an SDF (Structured-Data Format) file of all ligands in GtoPdb. SDF is a chemical-data file format developed by MDL. The format wraps individual ligands in molfile format and contains the structure connection table where we have a curated SMILES for the ligand (www.guidetopharmacology.org/DATA/all_ligands.sdf). The second file is a Ligand ID Mapping file. This contains GtoPdb ligand IDs mapped to equivalent external resource IDs. The file includes PubChem CID, PubChem SID, ChEMBL, ChEBI, UniProtKB, IUPAC, INN, CAS, DrugBank and DrugCentral identifiers (www.guidetopharmacology.org/DATA/ligand_id_mapping.csv).

### Database Links and interoperability

#### PubChem

**Figure 4.**
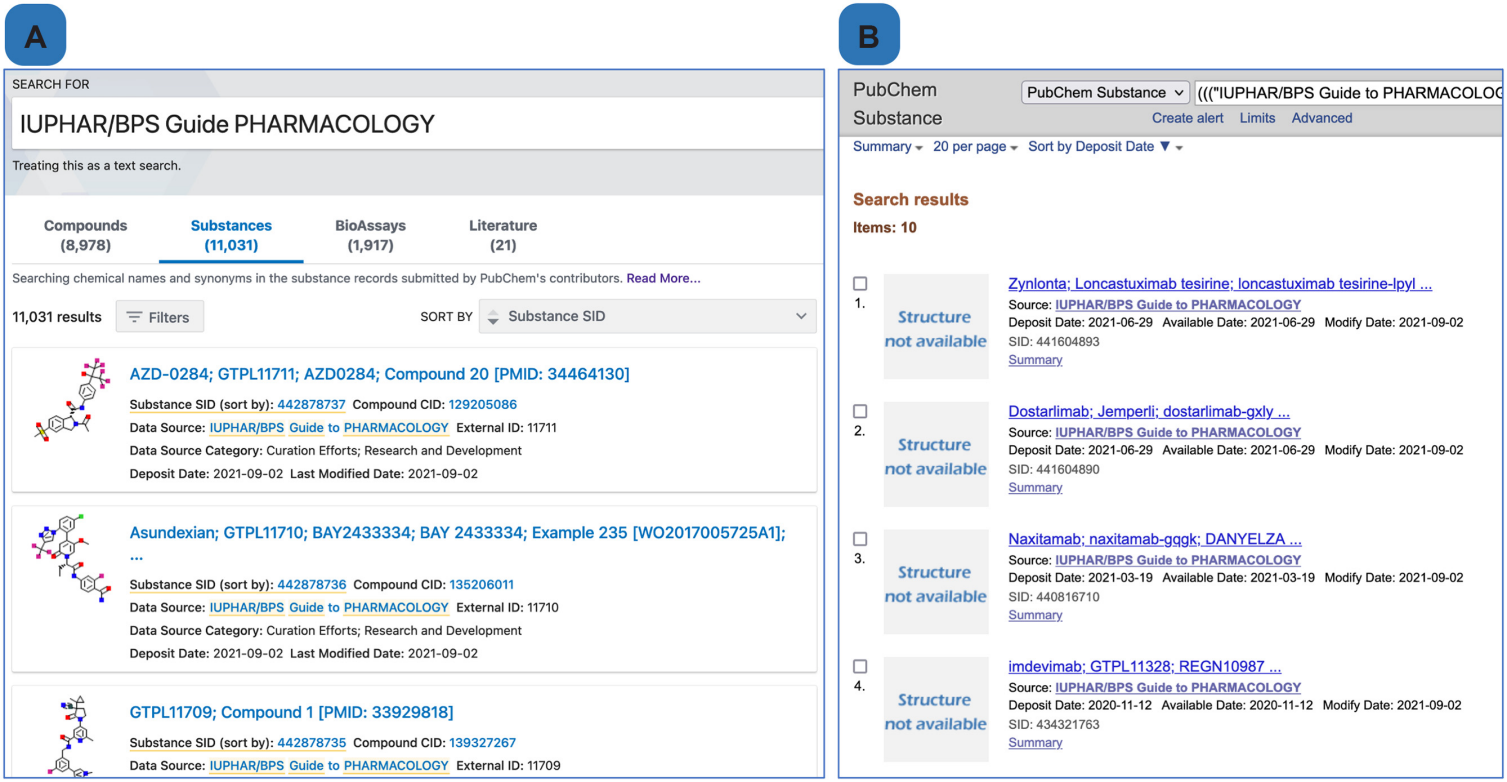
Guide to PHARMACOLOGY data in PubChem. Panel **A** shows the result of a main search on PubChem for ‘IUPHAR/BPS Guide to PHARMACOLOGY’. Panel **B** shows the result of searching PubChem substances for Guide to PHARMACOLOGY compounds, made available since 2020, tagged with our approved drug and antibody categories. Full query used is (((‘IUPHAR/BPS Guide to PHARMACOLOGY’[SourceName]) AND ‘gtopdb_approved’[Comment]) AND ‘gtopdb_antibody’[Comment]) AND (‘2020/01/01’[AvailableDate] : ‘3000’[AvailableDate]).

The powerful utilities arising from GtoPdb ligand data integration into PubChem (19) and other NCBI resources (20), have already been described (7). PubChem Substances are community-submitted compounds, and many may exist for the same molecule and each may contain different, submitter dependent, information about the molecule. PubChem extracts the unique chemical structures from Substance records (standardisation) and stores them as PubChem Compounds. This means that substance records from different data sources about the same molecule are aggregated in a common Compound record in PubChem. Using the PubChem Substance interface, the newest GtoPdb entries from the 2021.3 release have increased the Substance Identifier (SID) count to 11 031, as shown in Figure 4A via searching PubChem with the source term ‘IUPHAR/BPS Guide to PHARMACOLOGY’. This query also indicates that there is a PubChem Compound (CID) count of 8,978. The queries establish that we have an excess of SIDs which comprise ligand entities that are too large to form CIDs because they exceed the chemical structure specification upper limit of ∼ 70 amino acid or nucleotide polymeric units (our largest CID is agatolimod, an oligodeoxynucleotide TLR9 agonist with a MW of 7707). Thus, these 2053 SID-only ligands consist largely of antibodies, other protein ligands, plus larger peptides and polynucleotides.

We have been extending our ligand tagging to enable uses to retrieve sets of particular interest. Selections of five different tags are shown in Table 4. The approved drugs include 186 SID-only entries. Of these, 113 are antibodies, with the difference being large peptides or polynucleotides. Similarly, the immunopharmacology SIDs include 173 antibodies but the antimalarials just two, meplazumab and the CIS43 antibody.

**Table 4.** Counts of GtoPdb tagged ligands in PubChem (September 2021), using selected substance queries (column 2). CID numbers are retrieved via the ‘Find related data’ > ‘Database’ > ‘PubChem Compound’ > ‘PubChem Same Compound’ > CID count and display (i.e. a SID > CID conversion)

Ligand type	Query^a^	SID Count	CID count
Approved drugs	gtopdb_approved [comment]	1688	1502
Immunopharmacology	gtopdb_immuno [comment]	1345	928
Antimalaria	gtopdb_malaria [comment]	114	112
Antibacterial	gtopdb_antibacterial [comment]	311	311
Antibody	gtopdb_antibody [comment]	317	0

^a^Example query format for approved drugs ‘‘IUPHAR/BPS Guide to PHARMACOLOGY’[SourceName] AND ‘gtopdb_approved’[comment]’

Queries of our SID content can be combined as Boolean operations (as can any PubChem interface queries). The result shown in Figure 4B combines ‘approved drug’, ‘antibody’ and ‘available after 1 January 2020’.

The CID entries for GtoPdb can be browsed, interrogated, filtered and intersected (i.e. finding entries in common between other CID collections) with 100s of other data sources. PubChem has also recently enhanced their co-occurrence recommendations, which opens up another dimension to explore (21).

#### Comparison with other resources

As leading curated resources it is useful to compare GtoPdb with ChEMBL (18), BindingDB (22) and DrugBank (23) in terms of complementarity for users. While the data models and query functionality of the individual web sites are different, content comparison is made easier because not only have all four submitted to PubChem but they now also each have target cross-references in UniProtKB. The ligand and target counts and comparison with GtoPdb are shown in Table 5. Around 25% of GtoPdb compounds do not overlap with ChEMBL, largely due to divergent journal selection but also to the ∼12 month release cycle of the latter. In contrast GtoPdb releases 4–5 times a year. ChEMBL extracts all assay data, including ADMET determinations, from a paper whereas GtoPdb usually extracts just the lead compound but will also curate reported secondary target activity. BindingDB has a mirroring arrangement with ChEMBL from which it subsumes just the individual protein target-mapped data. It also uniquely curates SAR from patents with 379 307 compounds from 5082 US patents. While GtoPdb target overlap with both ChEMBL and BindingDB is extensive, Gtopdb has 209 not in ChEMBL and 319 not in BindingDB. An important difference between these three and DrugBank is that ligand-to-target binding data are not openly available due to DrugBank's commercial licencing conditions. Their compound overlap with GtoPdb of ∼30% is substantially lower than with ChEMBL and BindingDB. The difference in protein targets is also pronounced, with GtoPdb having only ∼50% overlap as UniProtKB cross-references. While DrugBank updates their website quarterly their latest PubChem submissions were from March 2020.

**Table 5.** Comparison of PubChem compound identifiers (CIDs) and UniProtKB protein identifiers between GtoPdb and ChEMBL, BindingDB and DrugBank

Resource	CID Total^a^	CID overlap with GtoPdb	Unique CID to GtoPdb	UniProtKB target total^b^	Target overlap with GtoPdb	Unique targets to GtoPdb
ChEMBL	2 085 502	6883	2091	9399	1850	202
BindingDB	1 017 955	5740	3234	8309	1735	317
DrugBank	11 205	2609	6365	5159	1047	1009

^a^CID counts are taken using the advanced PubChem Compound search (www.ncbi.nlm.nih.gov/pccompound), specifying source name in the query (i.e. ‘IUPHAR/BPS Guide to PHARMACOLOGY’[SourceName]) NOT ‘ChEMBL’[SourceName]).

^b^UniProtKB counts are taken from the UniProtKB advanced search, filtering on Cross-Reference > Chemistry Database (i.e. www.uniprot.org/uniprot/?query=database%3A%28type%3Aguidetopharmacology%29+database%3A%28type%3Abindingdb%29&sort=score).

#### External links

Since our last update, GtoPdb has added new out-links to several useful resources. Pharmacology-specific resources that have been added include DrugCentral (24), where we maintain links from our ligands, mapped to DrugCentral structure via InChi Key and PHAROS (17), where we link from GtoPdb targets via UniProtKB identifiers. Links have also been recently added from target pages to Alphafold (https://alphafold.ebi.ac.uk/) (25), which now means the majority of protein targets in GtoPdb link to a predicted 3D structure. Specialist links have been added, as already mentioned, to AntibioticDB and also to the RESOLUTE Knowledgebase (https://re-solute.eu/knowledgebase). RESOLUTE aims, through systematic and coordinated efforts, to improve understanding of the solute carrier (SLCs) proteins. These are a relatively understudied class of proteins and represent a largely untapped source of new potential drug targets. So building links between GtoPdb and RESOLUTE will bring benefits to users of both resources.

**Figure 5.**
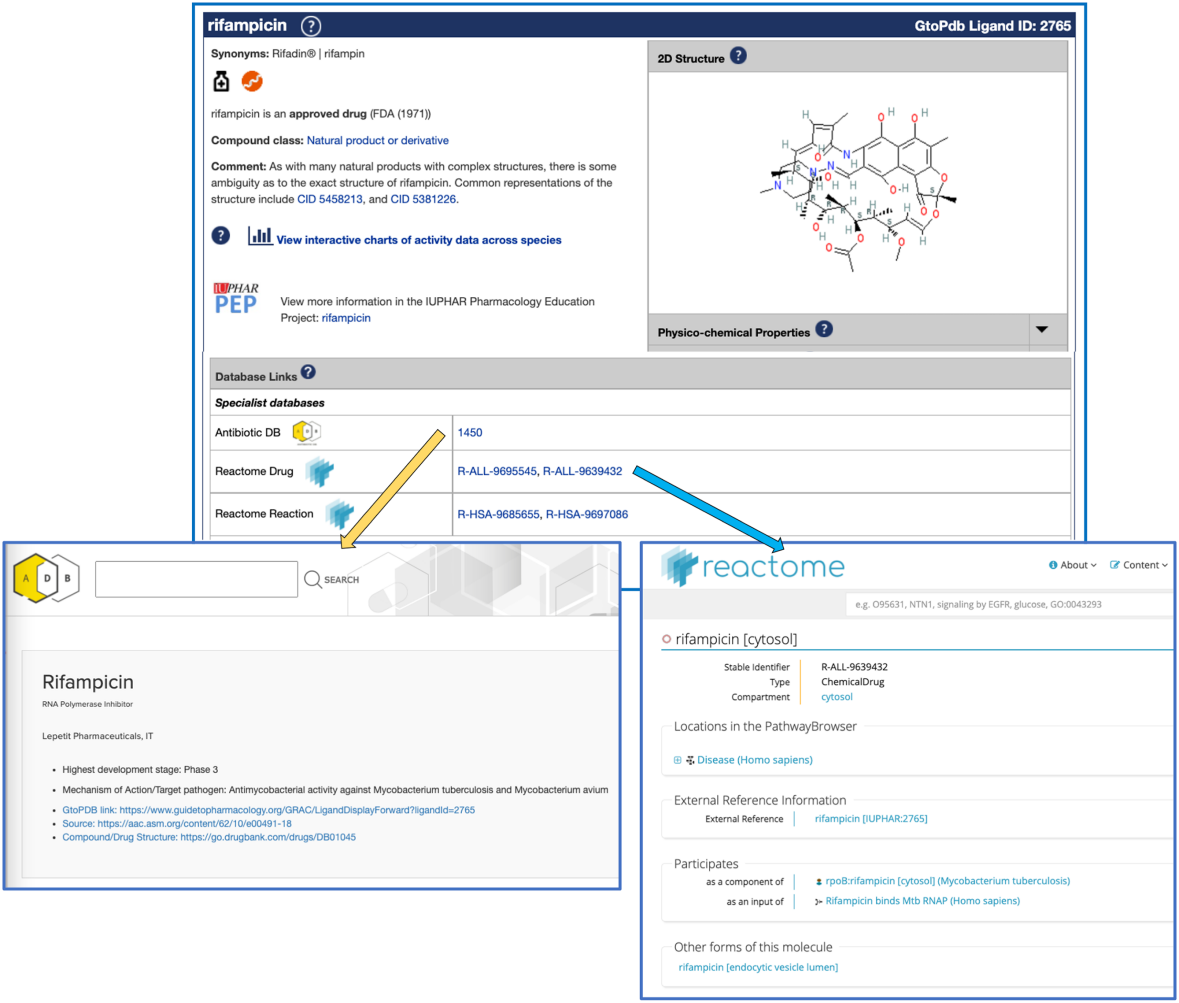
GtoPdb ligand summary page for rifampicin (www.guidetopharmacology.org/GRAC/LigandDisplayForward?ligandId=2765) highlighting new specialist database outlinks to AntibioticDB and Reactome.

GtoPdb has also established a strong engagement with Reactome (26) in the past year. Reactome already curates drugs/chemicals against Pathways and Reactions in their resource, and uses GtoPdb identifiers for drugs. They are in the process of using GtoPdb interaction data to allow further curation of drugs against reactions and pathways based on ligand interactions with proteins (via UniProtKB identifiers). Working collaboratively, we have mapped GtoPdb ligands to appropriate Reactome Drug and Reaction pages, and these links are now available on our ligand summary pages (Figure 5).

#### Bioschemas

The Guide to Pharmacology website now includes Bioschemas Schema.org mark-up on ligand and target pages and our coronavirus information page. BioSchemas (https://bioschemas.org) (27) aims to encourage life science resources to implement Schema.org mark-up onto their websites to make it simpler for search engines to index the website, and makes it easier to collate and analyze the data. We have focused on including mark-up on pages that detail ligands and protein targets

Initially, we focused on implementing mark-up on all ligand summary pages and target detail pages. This involves including properties from the Bioschemas MolecularEntity profile (https://bioschemas.org/profiles/MolecularEntity/0.5-RELEASE/) and Protein profile (https://bioschemas.org/profiles/Protein/0.11-RELEASE/). More recently, we have also added mark-up to the new coronavirus information page.

#### Collaborations and connectivity

Disseminating information about GtoPdb is done in several ways, including Social Media (Twitter (@GuidetoPHARM), Facebook and LinkedIn) to announce database updates, publications of interest and alert followers to upcoming events. Our blog is used to provide detailed descriptions of database releases, technical descriptions of developments, and commentaries on hot topics in pharmacology (https://blog.guidetopharmacology.org/category/hot-topics/). The Concise Guide to PHARMACOLOGY 2021/22 (28), published online in September 2021, is the fifth edition in a series of biennial publications which contain concise overviews of ∼1900 targets and ∼3500 ligands.

GtoPdb remains one of the ELIXIR-UK node services, under its human health and disease strategic theme (https://elixiruknode.org/human-health-and-disease/). Beyond collaborations already mentioned, GtoPdb also engages with Probes and Drugs (www.probes-drugs.org/home/), with one member of our group (CS) co-authoring a recent paper with them on probe compounds (29). They subsume each of our new releases integrating them with their other sources, they report that our ligand list includes 305 experimental probe compounds (see Table 9 in the reference) and we continue to collaborate with BindingDB for coronavirus protein targets to jointly curate selected papers and patents (www.bindingdb.org/bind/Covid19.jsp).

#### Immunopharmacology collaboration

The COVID-19 pandemic has underlined the crucial role of immunopharmacology in both research and teaching. A collaboration with the International Union of Immunopharmacology (IUIS) incorporated Immunopaedia (www.immunopaedia.org.za) into the Pharmacology Education Project (30) (www.pharmacologyeducation.org/) allowing worldwide education in immunological cell types, processes, drug targets and ligands for pharmacologists. This also strengthened curation into the emerging antibodies, and drugs in evaluation, for research purposes in the Guide to IMMUNOPHARMACOLOGY (www.guidetoimmunopharmacology.org).

#### Future directions

We will continue to evolve new techniques and metrics to measure the impact and value of the database for the global scientific community, and address new challenges. A significant recent development has been in the rapid increase in pre-prints that have not yet undergone peer review, particularly those reporting results for SARS-CoV-2 and COVID-19. Our objective will be to maintain a high-standard of curation by tracking the inclusion of pre-prints in the database and whether a subsequent reviewed paper is published or retracted.

Our strengths are incorporating recommendations for inclusion in the database from the thousands of scientists in our expert NC-IUPHAR subcommittees with manual curation, maximizing the value to database users by frequent updates. An immediate focus of future work is a project to add information about acute and chronic kidney injury that can be caused by a number of drugs used in routine clinical practice. Such drug-induced renal damage is a relatively common adverse event that contributes to morbidity and to significant healthcare costs.

We will continue to foster synergies with other databases to enhance our coverage in areas of pharmacology that have a global importance, as exemplified by Medicines for Malaria Venture and AntibioticDB.

#### Data access

GtoPdb, GtoImmuPdb and GtoMPdb are available online at https://www.guidetopharmacology.org, https://www.guidetoimmunopharmacology.org and https://www.guidetomalariapharmacology.org, respectively. All three resources are licensed under the Open Data Commons Open Database License (ODbL) (www.opendatacommons.org/licenses/odbl/) and the contents are licensed under the Creative Commons Attribution ShareAlike 4.0 International (CC BY-SA 4.0, https://creativecommons.org/licenses/by-sa/4.0/). Advice on linking to us and for accessing and downloading data are provided here: www.guidetopharmacology.org/linking.jsp. GtoPdb aims to make 3 to 4 public database releases per year; the data summaries and statistics reported in this paper are from release 2021.3 (September 2021). Our downloads page (available from www.guidetopharmacology.org/downloads.jsp) provides a dump file of the full PostgreSQL database, in addition to several specific download files for targets, ligands, interactions, peptides endogenous/natural ligands, and (new in 2021) approved rugs with primary targets, ligand ID mapping, and ligand as an SDF file. We also provide RDF flat files, which users can load into a local triple store and perform SPARQL queries across the data. Our REST web services are available at www.guidetopharmacology.org/webServices.jsp and provide computational access to data in JavaScript Object Notation (JSON) format. The web-services have been extended to include Guide to Malaria Pharmacology filters on ligands, targets and interactions. We encourage users to communicate with us if they download data in any format, both for further advice and to be aware of applications using GtoPdb data.

#### Citing the resource

This publication replaces all previous papers for citing this resource. Citation advice for specific target pages appears on the website. Please refer to our resources on first mention by full correct name (IUPHAR/BPS Guide to PHARMACOLOGY, IUPHAR Guide to IMMUNOPHARMACOLOGY, IUPHAR/MMV Guide to MALARIA PHARMACOLOGY), including the capitalization. For subsequent abbreviation, please use GtoPdb, GtoImmuPdb and GtoMPdb, specifying the release version number (this can be found on our About page - www.guidetopharmacology.org/about.jsp#content).

## Supplementary Material

gkab1010_Supplemental_File

## Notes

Present address: Christopher Southan, Medicines Discovery Catapult, Alderley Park, Macclesfield SK10 4ZF, UK.

Present address: Adam J. Pawson, Bitesize Bio, Science Squared Limited, Gorebridge EH23 4TT, UK.
