# Supplementary material for: The IUPHAR/BPS guide to PHARMACOLOGY in 2022: curating pharmacology for COVID-19, malaria and antibacterials
Source: Nucleic Acids Res. 2021 Oct 30;50(D1):D1282–94. doi: 10.1093/nar/gkab1010 (PMC8689838; doi:10.1093/nar/gkab1010)
Supplement: gkab1010_Supplemental_File [file gkab1010_supplemental_file.docx]

| **Ligand Name** | **Ligand ID** | **URL** |
| --- | --- | --- |
| bemnifosbuvir | 11295 | <https://www.guidetopharmacology.org/GRAC/LigandDisplayForward?ligandId=11295> |
| lufotrelvir | 11249 | <https://www.guidetopharmacology.org/GRAC/LigandDisplayForward?ligandId=11249> |
| molnupiravir | 10737 | <https://www.guidetopharmacology.org/GRAC/LigandDisplayForward?ligandId=10737> |
| eclitasertib | 11308 | <https://www.guidetopharmacology.org/GRAC/LigandDisplayForward?ligandId=11308> |
| enpatoran | 11307 | <https://www.guidetopharmacology.org/GRAC/LigandDisplayForward?ligandId=11307> |
| PF-07321332 | 11503 | <https://www.guidetopharmacology.org/GRAC/LigandDisplayForward?ligandId=11503> |
| imdevimab | 11328 | <https://www.guidetopharmacology.org/GRAC/LigandDisplayForward?ligandId=11328> |
| casirivimab | 11327 | <https://www.guidetopharmacology.org/GRAC/LigandDisplayForward?ligandId=11327> |
| remdesivir | 10715 | <https://www.guidetopharmacology.org/GRAC/LigandDisplayForward?ligandId=10715> |
| baricitinib | 7792 | <https://www.guidetopharmacology.org/GRAC/LigandDisplayForward?ligandId=7792> |
| agatolimod | 9843 | <https://www.guidetopharmacology.org/GRAC/LigandDisplayForward?ligandId=9843> |
| meplazumab | 11026 | <https://www.guidetopharmacology.org/GRAC/LigandDisplayForward?ligandId=11026> |
| CIS43 antibody | 11254 | <https://www.guidetopharmacology.org/GRAC/LigandDisplayForward?ligandId=11254> |
|  |  |  |
| **Target Name** | **Target ID** | **URL** |
| H_1_ receptor | 262 | <https://www.guidetopharmacology.org/GRAC/ObjectDisplayForward?objectId=262> |
| 5-HT_2C_ receptor | 8 | <https://www.guidetopharmacology.org/GRAC/ObjectDisplayForward?objectId=8> |
|  |  |  |
| **Family Name** | **Family ID** | **URL** |
| Coronavirus (CoV) proteins | 1034 | <https://www.guidetopharmacology.org/GRAC/FamilyDisplayForward?familyId=1034> |
| Antimalarial targets | 970 | <https://www.guidetomalariapharmacology.org/GRAC/FamilyDisplayForward?familyId=970> |

Table S1. Table of ligand, proein target and protein target family names, IDs and URLs referred to in the paper
